# Determination of Ischemia Onset Based on Automatically Generated Spectralis SD-OCT Values in Acute Central Retinal Artery Occlusion

**DOI:** 10.1155/2021/5527292

**Published:** 2021-04-14

**Authors:** Maria Casagrande, Robert Kromer, Daniel A. Wenzel, Sven Poli, Martin S. Spitzer, Vasyl Druchkiv, Maximilian Schultheiss, Spyridon Dimopoulos

**Affiliations:** ^1^Department of Ophthalmology, University Medical Center Hamburg-Eppendorf, Hamburg, Germany; ^2^University Eye Hospital, Centre of Ophthalmology, University Medical Center Tübingen, Tübingen, Germany; ^3^Department of Neurology & Stroke, University Medical Center Tübingen, Tübingen, Germany; ^4^Hertie Institute for Clinical Brain Research, University Hospital Tübingen, Tübingen, Germany

## Abstract

Acute central retinal artery occlusion (CRAO) induces a time-dependent increase in retinal thickness. By manually measuring the relative retinal thickness increase (RRTI) in comparison to the contralateral eye based on optical coherence tomography (OCT), ischemia onset within the past 4.5 hours could be determined with 100% sensitivity and 94.3% specificity. To enable examiner-independent and quicker diagnostics, we analyzed the RRTI using the automatic retinal thickness measurement. In this retrospective study, 28 eyes were evaluated with an acute CRAO (<46 hours). All patients received a Spectralis SD-OCT image of both eyes. The RRTI was calculated for the ETDRS sectors using the Segmentation Module for Single Retinal Layer Analysis. Receiver operating characteristic (ROC) analysis was performed to determine patients ≤4.5 hours by RRTI. In all sectors, time to OCT (TTO) and RRTI correlated positively. The optimal cutoff point to detect CRAOs ≤4.5 hours was between 18.7% nasally and 22.9% RRTI temporally. Sensitivity and specificity varied between the sectors with 90–95% sensitivity and 89–100% specificity. In conclusion, the automatic measurement of RRTI also allows the differentiation of CRAOs within a possible therapeutic time window ≤4.5 hours and CRAOs ≥4.5 hours with a high sensitivity and specificity. Additionally, it offers quicker, easier, and a user-independent assessment of ischemia onset, helping to set a base for establishing automatic indices generated by the OCT machines.

## 1. Introduction

Acute central retinal artery occlusion (CRAO) is an ophthalmic emergency causing massive monocular vision loss. An evidence-based therapy does not exist, but recent case series and meta-analyses show that intravenous fibrinolysis administered within 4.5 hours could enhance the prognosis of CRAO patients [1–3]. Determining the onset of CRAO is sometimes difficult. Only if monocular sudden vision loss is recognized with both eyes open, the patients can exactly determine the time of symptom onset. One-third of the CRAO cases occur during nocturnal sleep [4]. Other patients recognize the vision loss by accidentally closing the unaffected eye and consequently report a wrong time of symptom onset. If the onset of ischemia is unsure, patients will not qualify for treatment options as risks outweigh benefits. Determining the onset of ischemia is therefore crucial to introduce patients to therapeutic options.

In a previous study [5], we have shown that, in CRAO patients, ischemia onset ≤4.5 hours can be determined by manual measurement with a sensitivity of 100% and a specificity of 94.3%. This manual analysis of optical coherence tomography (OCT) scans is based on the relative retinal thickness increase (RRTI) in comparison to the fellow eye. As the central retinal artery (CRA) provides the blood flow solely for the inner retinal layers (IRL; ganglion cell layer, inner plexiform cell layer, inner nuclear layer, and outer plexiform layer), retinal thickness increase generally results from IRL edema [6–13]. Over time, in the chronic phase of CRAO, the edema will eventually resolve and be followed by retinal atrophy in the IRL [8, 12–15]. As the possible therapeutic time window is approximately 4.5 hours and the chances for a functional recovery increase with shorter time of ischemia, any diagnostic test needs to be quick and easy to perform. Ideally, this analysis is performed user independent in an automated manner by the respective OCT device. In this study, automatic values from Spectralis SD-OCT were used to calculate RRTI in order to provide a quick, easy, and examiner-independent analysis of ischemia onset in a CRAO patient.

## 2. Methods

### 2.1. Study Design and Patient Selection

In this retrospective study, data of 28 patients diagnosed with CRAO between January 2010 and December 2019 from the departments of ophthalmology at the University Medical Center Hamburg-Eppendorf, Germany, and Eberhard Karls University in Tübingen, Germany, were evaluated. Inclusion criteria were reliable patient-reported time of symptom onset and an OCT scan covering all ETDRS sectors of both eyes performed at a primary visit within 48 hours after ischemia onset (time to OCT, TTO). Reliable patient-reported time of symptom onset was assessed according to a previous study on lysis by our research group [2]. Exclusion criteria were anisometropia of more than 3 diopters, retinal pathologies other than CRAO (e.g., macular degeneration and epiretinal gliosis), a cilioretinal artery, hemi-CRAO, and a reperfused CRAO. Exclusion criteria apply to both the study and the control eye.

According to the local ethics committee (Hamburg, Germany), approval and written patient consent were not necessary as the retrospective data were anonymized at the source, and data were collected from routine clinical diagnostics.

### 2.2. Optical Coherence Tomography-Based Calculation of Relative Retinal Thickness Increase

OCT scans were performed with spectral-domain OCT (Spectralis OCT; Heidelberg Engineering, Germany). A volume scan of both eyes (the affected and unaffected eye) was analyzed. Grid type was 1, 3, and 6 mm ETDRS. Scans were checked for a straight alignment through the fovea. Data were calculated automatically using the Spectral-Domain Optical Coherence Tomography Segmentation Module for Single Retinal Layer Analysis. The retinal thickness of the unaffected eye (control) served as the baseline as it was assumed to be identical to the eye with CRAO before ischemia onset. Absolute retinal thickness change is the difference between the maximum retinal thickness of the affected eye and the baseline. The RRTI is the increase in retinal thickness of the affected eye compared to the unaffected eye in percent. Manual measurement of the retinal thickness was performed like previously described [5]. In brief, the thickest point of the retina in the papillomacular bundle was manually measured with the virtual ruler tool of Heidelberg Eye Explorer software.

### 2.3. Statistics

To investigate the association between metric variables and time to OCT since symptom onset, linear regression was employed within the first 48 hours. When dividing patients into two groups with a TTO of ≤4.5 hours (positives) and >4.5 hours (negatives) and using metric variables as a marker for classification between these groups, receiver operating characteristic (ROC) analysis was performed. ROC analysis graphically illustrates the diagnostic ability of optical coherence tomography- (OCT-) based relative retinal thickness increase (RRTI) to distinguish between time to OCT of less and more than 4.5 hours. This was performed for all 4 ETDRS sectors. The ROC curve is created by plotting true positive values (sensitivity) against false positive values (1 − specificity). The optimal cutoff point was chosen by maximizing the sum of sensitivity and specificity. A sensitivity of 100% means that all CRAOs within the time frame of 4.5 hours were identified. A specificity of 100% means that all CRAOs ≥4.5 hours were excluded.

All calculations were done with R Core Team (Vienna, Austria, 2019). Parametric *t*-tests were applied, and *p* values were two-tailed. Statistical significance was set at *p* < 0.05.

## 3. Results

### 3.1. Relative Retinal Thickness Increase

**Figure 1:**
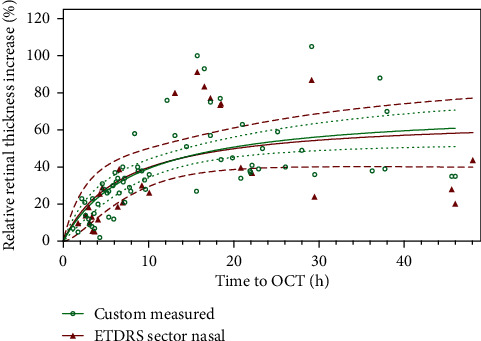
Representative time-dependent increase of the relative retinal thickness over 48 hours compared between manual and automatic measurements in the nasal sector. Relative retinal thickness increase (RRTI) in central retinal artery occlusion (CRAO) is caused by retinal ischemia. This figure shows the nasal ETDRS sector (*n* = 28) represented by the triangle and manually measured values from the previous study [5] represented by circles (*n* = 66). The *x*-axis represents time to OCT (TTO) and the *y*-axis the relative retinal thickness increase in percent.

RRTI was measured between 1.75 and 48.0 hours after ischemia onset and differed between the ETDRS sectors. In all sectors, TTO and RRTI correlated positively.  Figure 1 displays this exemplary for the nasal sector over the full time frame of 48 hours for both the manual [5] as well as the automatic measurements. The RRTI calculated with either measurement tool (manual or automated, *p* = 0.8372) follows a hyperbolic curve with a steep incline in the first 10 hours and then reaching a plateau.

**Figure 2:**
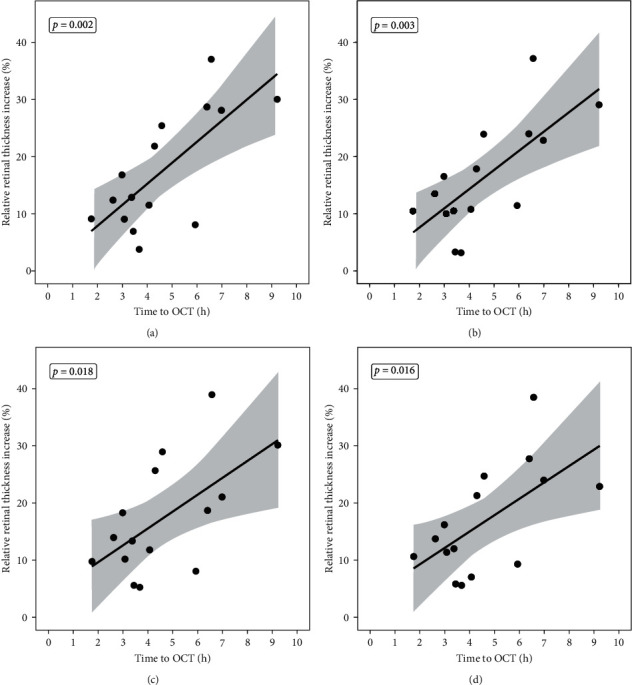
Time-dependent increase of the relative retinal thickness over 10 hours. The figure displays the RRTI in all ETDRS sectors: (a) inferior, (b) superior, (c) nasal, and (d) temporal (*n* = 15). In every figure, the *x*-axis represents time to OCT (TTO) and the *y*-axis the RRTI in percent in the respective sectors. Every dot represents an analyzed patient. The straight line is the ordinary least squares (OLS) regression over the first 10 hours after ischemia onset. The grey region around the regression line describes the 95% confidence interval for the expected value of *y* given *x*. It reflects the confidence in estimated parameters.

In Figure 2, all ETDRS sectors are represented for the first 10 hours. During that time, the correlation between TTO and the respective ETDRS was statistically significant (nasal: *p* = 0.018; superior: *p* = 0.003; temporal: *p* = 0.016; inferior: *p* = 0.002). The highest statistical significance in correlation was therefore in the inferior sector and the lowest in the nasal sector.

### 3.2. Sensitivity and Specificity

OCT-based retinal thickness analysis was used to predict the potential therapeutic time window ≤4.5 hours after the ischemic incident by evaluating it by multiple logistic regression analysis using RRTI as the independent variable. Receiver operating characteristic (ROC) curve analysis was used to display true positive and false positive rate.

**Figure 3:**
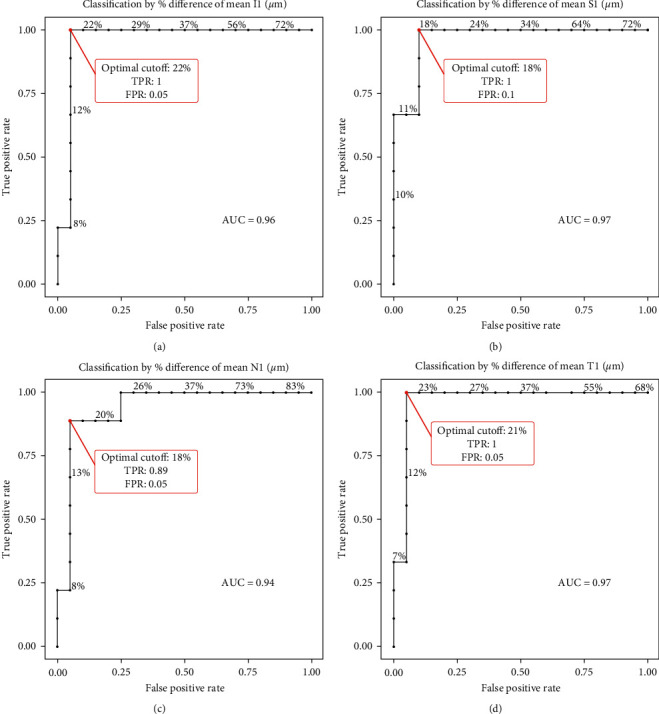
Receiver operating characteristic (ROC) curve analysis. Calculation of the diagnostic performance of optical coherence tomography- (OCT-) based relative retinal thickness increase (RRTI) to distinguish between time to OCT of less and more than 4.5 hours in all 4 ETDRS sectors. The inferior sector (a), the superior sector (b), the nasal sector (c), and the temporal sector (d). On the *y*-axis, the true positive value represents sensitivity, while the *x*-axis shows false positive values from which the specificity can be calculated by 1 − false positive value. The area under the ROC curve (AUC) is given for each graph.

The area under the curve (AUC) differed between the sectors from 0.94 (nasal) and 0.96 (inferior) to 0.97 (superior and temporal) (Figure 3).

The optimal cutoff point, where sensitivity and specificity are maximized, varied between the sectors with higher sensitivity than specificity in the superior, temporal, and inferior sector. In the nasal sector, specificity was higher than sensitivity. Inferiorly, the optimal cutoff point was 21.8% (100% sensitivity; 95% specificity), superiorly 17.8% (100% sensitivity; 90% specificity), nasally 18.3% (89% sensitivity; 95% specificity), and temporally 21.3% (100% sensitivity; 95% specificity).

## 4. Discussion

**Figure 4:**
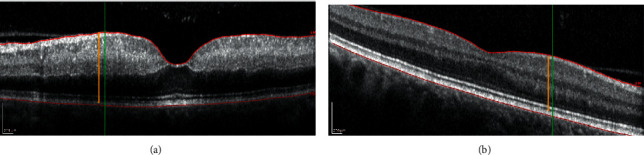
Automatic versus manual determination of retinal thickness representative optical coherence tomography (OCT) scan for the affected eye (a) and the unaffected eye (b). The scan in the unaffected eye is diagonal. Measurements are performed manually (orange line) and automatically (green line). The manual measurement equals 466 *µ*m (a) and 335 *µ*m (b), while the automatic is 474 *µ*m (a) and 377 *µ*m (b). The absolute retinal thickness increase (ARTI) is therefore different: 1.26 in the manual measurement and 1.39 in the automatic.

In previous studies, we presented the temporal changes of retinal thickness in acute CRAO patients [5, 16]. We showed with a sensitivity of 100% and a specificity of 94.3% that RRTI is a valuable surrogate parameter to determine if ischemia onset was within the last 4.5 hours [5]. In our previous publication, the total retinal thickness was measured manually at the thickest point between the optic nerve and fovea, and this measurement was always performed perpendicular to the retinal pigment epithelium (RPE). In the automatic analysis of the present study, the measurement is performed vertically to the screen and not perpendicular to the RPE. This might lead to a more accurate measurement in the manual analysis (Figure 4).

In contrast, in this study, four different parafoveal sectors of the retina were evaluated and not only a single point at the retina, which in turn might lead to more robust and objective results. Furthermore, automatic measurements could be performed by optometrists, technicians, or physician assistants if patients enter primary care facilities with acute visual loss. Also, they could be the base for machine-generated indices. These would make identification of IVT-eligible CRAO patients quick, easy, and user independent to perform.

Comparing the manual and automated measurements, as displayed in Figure 1, it was shown that, under both measurement conditions, RRTI followed the same increase and plateau phase. This supports the fact that both methods are on par regarding the determination of ischemia onset.

Concerning sensitivity and specificity to detect CRAO patients ≤4.5 hours since symptom onset, the results vary in between the ETDRS sectors (sensitivity/specificity: inferior: 100%/95%; superior: 100%/90%; nasal: 89%/95%; temporal: 100%/95%) and the manual assessment (sensitivity: 100%; specificity 94.3%) [5]. Fortunately, for both—the manual and the automatic measurement—high sensitivity and specificity could be achieved [5]. In manual measurements, the cutoff RRTI was 24.5% [5], while in the automatic measurements, it varied between 18.7 and 22.9%. The optimal cutoff point is so far unclear but is assumed to be between 18 and 25% to distinguish between CRAO patients ≤4.5 hours and ≥4.5 hours. Nevertheless, this does not mean that this cutoff is also the right cutoff for a good clinical outcome after IVT. This will have to be evaluated in multicenter placebo-controlled trials, e.g., the Early REperfusion Therapy with Intravenous Alteplase for Recovery of VISION in Acute Central Retinal Artery Occlusion (REVISION) trial starting soon in Germany or the THEIA trial in France.

Our study has some limitations as the retrospective character. Additionally, due to the fact that many patients analyzed in our previous study (*n* = 66) had a too small grid for covering all ETDRS sectors, the number of included patients into this study is lower than in our previous study. Furthermore, there is a selection bias of the included patients as we could only include patients with a certain symptom onset. Another limitation is that only patients with healthy eyes prior to CRAO were included as other retinal diseases on the affected or unaffected eye could influence the analysis.

Like in our previous study, the retinal thickness increases in a near-linear progression within the first hours, and consequently, CRAO onset can be estimated to a certain extent [5]. Determining the ischemia onset might be crucial in future because studies and meta-analyses report promising results for intravenous thrombolysis (IVT) if it is applied within the first 4.5 hours after ischemia onset [1–3, 17]. In ischemic stroke patients with unknown symptom onset, magnetic resonance imaging (MRI) can be performed to evaluate if those patients are eligible for reperfusion therapy or not. A multicenter, randomized, placebo-controlled study (WAKE-UP trial) reported that, in ischemic stroke patients, the outcome was significantly better compared to the placebo group if IVT is administered according to the MRI results. We discussed this in detail in the previous study [5]. In analogy to the MRI in ischemic stroke patients, RRTI from OCT measurements might be used in CRAO patients with unknown symptom onset.

**Figure 5:**
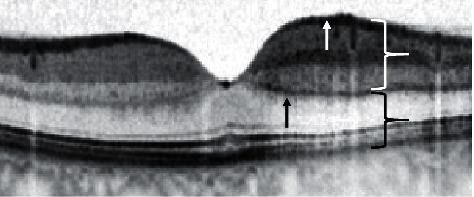
Signs of acute retinal ischemia in optical coherence tomography. This figure displays an optical coherence tomography (OCT) scan of a branch retinal artery occlusion with a time of symptom onset <2 hours. On the left side of the fovea, the retinal perfusion is intact, and the structure of the retinal layers is physiological. On the right side, where the branch retinal artery occlusion occurred, signs of ischemia can be seen in the scan. The white curly brace shows the hyperreflectivity in the inner layers of the retina. Consequently, the outer retinal layers and the retinal pigment epithelium show a hyporeflectivity—pointed out by the black curly brace. The black arrow points to the prominent middle-limiting membrane sign (pMLM). The white arrow symbolizes the thickening of the retina in comparison to the nonaffected part of the retina on the left side.

Besides being a possible surrogate parameter to determine eligibility for reperfusion therapy, OCT can be used to document ischemic changes in the inner part of the retina. At an early stage after the ischemic incident, before funduscopic changes such as the cherry red spot are visible, OCT demonstrates alterations. The inner retinal layers thicken and become more hyperreflective, while the outer retinal layers become less reflective due to the shadow effect of the inner retinal layers. Additionally, a demarcation line might form at the border of retinal and choroidal blood supply at the level of the outer plexiform layer (Figure 5). This phenomenon is known as the prominent middle-limiting membrane sign (pMLM), which is a pathognomonic sign for ischemia in the inner part of the retina [18].

In conclusion, the automatic analysis of retinal thickness is in line with our previously reported results. Even with the RRTI based on an automatic measurement of retinal sectors, CRAO patients with less than 4.5 hours since symptom onset are detected with a very high sensitivity and specificity. Nevertheless, these results are preliminary, and the RRTI as a surrogate parameter for good clinical outcome after IVT will be further analyzed in the REVISION trial. If placebo-controlled trials (such as the THEIA study, France, and the REVISION trial, Germany) confirm IVT as an evidence-based therapy within the first 4.5 hours after CRAO onset, determining the ischemia onset will be crucial, and automated OCT analysis might be the tool of choice to evaluate IVT eligibility.

## Data Availability

The data that support the findings of this study are available from the corresponding author (MS) upon reasonable request.
